# High lead exposure in two leaded bronze ingot foundry workers

**DOI:** 10.1186/s40557-014-0038-8

**Published:** 2014-12-01

**Authors:** Yoojun Song, Chunhui Suh, Shin-Ae Kim, Nami Kim, Sung-Min Kim, Seong-Wook Jeong, Se-Yeong Kim, Kun-Hyung Kim, Jeong-Ho Kim, Byung-Chul Son, Chae-Kwan Lee, Jong-Tae Lee

**Affiliations:** Department of Occupational and Environmental Medicine & Institute of Environmental and Occupational Medicine, Busan Paik Hospital, Inje University, 75, Bokji-ro, Busanjin-gu, Busan, 633-165 Republic of Korea

**Keywords:** Foundry, Lead exposure, Leaded bronze ingot

## Abstract

**Background:**

Awareness about lead poisoning in South Korea has increased; however, occupational exposures occurring in small-scale businesses have not been thoroughly investigated. We report two cases of high lead exposure in a leaded bronze ingot foundry.

**Case presentation:**

Two employees, a 54-year-old primary operator and a 46-year-old assistant, at a small-scale metalworking company who had been employed for 18 years and 1 month, respectively, showed elevated blood lead levels (61.1 μg/dL and 51.7 μg/dL, respectively) at an occupational health checkup. Neither worker complained of abnormal symptoms nor signs related to lead poisoning. Health assessment follow-ups were conducted and biological exposure indices of lead were calculated every four weeks. After the initial follow-up assessment, both workers were relocated from the foundry process to the metalworking process. In addition, a localized exhaust system was installed after the second follow-up.

**Conclusions:**

Foundry workers in a small-scale businesses might be at high risk of lead exposure because these businesses might be vulnerable to poor industrial hygiene. Therefore, regular occupational health checkups are required.

## Background

Bronze is an alloy consisting primarily of copper, but also contains other elements including manganese and aluminum. Leaded bronze (bronze containing lead) increases the plasticity of bronze, simplifies product fabrication, helps retain favorable thermal conductivity, and helps maintain good lubricity [1,2]. As a result, leaded bronze is used for fabrication processes that require precision. When a leaded bronze ingot is melted at high temperatures of 500°C–600°C, lead fumes are created as a by-product. The melting point of lead (327.46°C) is relatively lower than that of other substances; therefore, lead easily sublimates, even at particularly low temperatures. For these reasons, the melting and casting of leaded bronze ingots can result in lead exposure, which occurs as the lead fumes spread throughout the workplace.

The distribution of inhaled lead fumes in the human respiratory tract depends on particle size (typically <5 μm can be damaging), the anatomy of the respiratory tract, mucociliary clearance, and breathing habits [3]. In cases of poor personal hygiene, frequent cigarette smoking, or poor environmental conditions, lead fumes can be absorbed orally by the human body, and lead intake can cause lead poisoning in some individuals. Acute or chronic symptoms of lead poisoning can manifest in multiple systems of the body including the central and peripheral nervous systems, erythropoietin system, cardiovascular system, musculoskeletal system, immune system, and/or reproductive system [4].

When lead absorbed by the human body enters the bloodstream, the blood lead level (BLL) rises. Several guidelines for BLL monitoring have been proposed. The Occupational Safety and Health Administration recommends that individuals avoid tasks that lead to further lead exposure if their BLL is ≥60 μg/dL (or reaches a mean level of 50 μg/dL on three or more tests) [5]. In South Korea, guidelines for periodic occupational health checkups recommend reducing lead exposure if the BLL is ≥30 μg/dL and that these individuals receive a checkup every 2 months until two consecutive BLLs readings are <30 μg/dL [6].

Reported cases of occupational lead poisoning in South Korea include lead battery poisoning (1972 and 1986), the Janghang Smelting lead poisoning incident (1978), and the Banwol Industrial Complex lead poisoning incident (1983) [7-9]. Since these incidents, legal regulations and policies have been strengthened and regular measurements of workplace environmental levels and health examinations have been performed; as a result, the incidence of lead poisoning has decreased [10-12]. However, incidences of lead poisoning have still been reported in a small-scale businesses with long-term employees because legal management is difficult [13]. To our knowledge, no reported cases of high-lead exposure among leaded bronze ingot foundry workers are present in South Korea. Here, we present two cases with increased BLLs after working in a leaded bronze ingot foundry.

## Case presentation

### Occupational and medical history

The first patient was a 54-year-old man with 18 years of experience working in a foundry as a primary operator. He had been employed at a hydraulic equipment manufacturing company for the past 10 years and worked at the melting furnace and in molding frame department. He had a history of hyperthyroidism and smoked five pack-years, but had no family history of disease.

The second patient was a 46-year-old man with seven, three, and seven years of experience plating, driving a taxi, and driving a truck, respectively. He had been employed at the same manufacturing company as an assistant operator at the melting furnace and in molding frame department for one month. He had no history of illness or any hereditary diseases, but smoked 20 pack-years.

Neither patient had a history of herbal or health supplement intake. During the initial occupational health checkup, neither patient complained of abnormal symptoms such as muscle weakness, paresthesia, abdominal colicky pain, nausea, vomiting, diarrhea, or constipation. No abnormalities in the heart or respiratory sounds were noted upon auscultation. In addition, no tenderness or distension was observed during the abdominal examination. Moreover, no indications of Burton’s lines around the gums were present, and neither patient showed signs of pallor, petechiae, dyspnea on exertion, fatigue, malaise, or other symptoms related with anemia.

### Workplace evaluation

The small-scale, hydraulic machine parts manufacturing company that employed these two patients also employed 17 other workers (19 employees in total). After industrial materials were received at the foundry, they were subjected to the melting and molding processes, heat treatment, rough grinding, and the finishing process using a computerized numeral control (CNC) and machining center-tooling system. Both patients were involved in the melting and molding processes that required pre-heating the furnace for approximately 2.5 h from 6:30 AM to 9:00 AM and melting the primary material while simultaneously using reducing agents; these steps are performed to improve machinability (through higher plasticity), thermal conductivity, and lubricity. The primary material handled was a leaded bronze ingot (9%–11% lead content), and the company had an inspection certificate for the primary material (Table 1). Two different powder reducing agents were used. The first powder contained carbon that was used to maintain the oxygen level and temperature of the molten metal as well as block any moisture. The second powder contained a mixture of borax and ammonium chloride, an inorganic flux, which was used to maintain the product’s vacuum condition and lower the melting point of the primary material [14]. The company did not have a Material Safety Data Sheet (MSDS) for the reducing agents. The results of chemical composition analysis of the primary material and reducing agents are shown in Table 1.

**Table 1:** **Chemical composition of raw materials**

	**Chemical composition** **(%)**			
	**Base material**		**Reducing agent**	
	**Inspection certificate**	**Bulk sampling**	**Bulk sampling** * **Carbon**	Bulk sampling* Borax
Cooper	77.0–81.0	79.950	0.189	0
Tin	9.0–11.0	9.320	0	0
Lead	9.0–11.0	10.310	0.203	0
Zinc	−1.0	0.020	0.029	0
Iron	−0.2	0.010	0.050	0
Nickel	−1.0	0.290	0.020	0
Antimony	−0.5	0.090	0	0
Phosphorus	−0.05	0.009	0	0
Aluminium	−0.005	0	0.007	0.036
Silicon	−0.005	0	0	0

*Bulk sampling refers to the collection of the base material in the workplace and the laboratory analysis of these substances.

**Figure 1:**
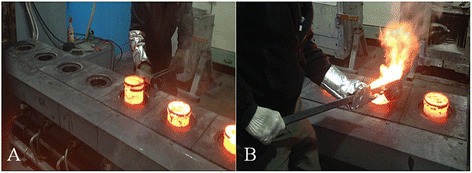
**Pouring molten metal into a molding frame.** **(A)** Setting of the molding frame by the assistant operator. **(B)** Pouring the molten metal into the molding frame by the primary operator.

After the leaded bronze ingot was melted for 1–1.5 h, the molten metal was inserted into a molding frame for 5–10 min. This task was repeated five to six times per day. While the assistant operator prepared the preheated molten frame (Figure 1A), the primary operator would scoop the molten metal from the furnace and insert it into the molding frame (Figure 1B).

The area in the foundry where the melting furnace and molding frames were set is isolated from other metalworking process areas. At the time of the initial occupational health checkup, the exhaust system used in the foundry consisted of an open canopy hood above the furnace. As such, the outlet for the exhaust air from the precipitator was located inside the foundry. Therefore, hazardous substances absorbed by the exhaust system might have been reintroduced into the foundry. The furnace was replaced every 2 months. However, we found an expended furnace in the furnace room that had been disassembled. The chemical composition analysis of the furnace revealed concentrations of lead, nickel, tin, and copper at 1.135%, 0.039%, 0.028%, and 4.112%, respectively. Respiratory protection masks (level 2 dusk masks, 3 M, 8710 L) were sometimes worn, but were usually removed when they became uncomfortable. The results of the work environment assessment are shown in Table 2. The airborne and personal lead concentrations of the melting furnace and molding frame were below the occupational exposure limit.

**Table 2:** **Measurements in the work environment**

**Measured results** **(mg/** **m** ^**3**^ **)**	**OEL** **(TWA, ** **mg/** **m** ^**3**^ **)**	**Initial measurement December 9, ** **2013**		**Follow** **-up measurement February 4, ** **2014**			
		**Primary operator**	**Assistant operator**	Primary operator*	Assistant operator*	**Area sample of the melting furnace**	**Area sample of the molding frame**
Lead	0.05	0.0073	ND	0.0013	0.0394	0.0310	0.0225
Nickel	0.5	0.0003	ND	0.0002	0.0001	0.0001	ND
Tin	2	0.0036	0.0002	ND	ND	ND	ND
Cooper	1	0.0006	0.0003	0.0032	0.0013	0.0009	ND

*ND*, not detectable; *OEL*, occupational exposure limit; *TWA*, time-weighted average.

*New workers were assigned after the primary operator and his assistant were transferred out of the foundry.

After the first follow-up following the initial checkup, the two patients were removed from the foundry and relocated to the metalworking process (January 20, 2014). After the second follow-up, a localized exhaust system was installed (March 20, 2014); slot hood over the molding frame, enclosed canopy hood over the furnace and outside movement of the exhaust air outlet. Soon thereafter, the assistant operator left the company (March 24, 2014); hence, the fourth follow-up on this assistant was not conducted.

### Health assessment and biological exposure indices

After the initial occupational health checkup, follow-up assessments were conducted every four weeks. The follow-up results of each patient are shown in Table 3. At the initial checkup, both the primary operator and assistant operator had an elevated BLL of 61.1 μg/dL and 51.7 μg/dL, respectively. However, at the first follow up, the BLL of the assistant (63.1 μg/dL) was higher than that of the primary operator’s was (68.8 μg/dL). After the patients had been relocated from the foundry to the metalworking process, the BLL of the assistant decreased at a slower rate than that of the primary operator did according to the results of the second and third follow-ups. At the initial blood test, the assistant had an increased white cell count of 10,090 μL, but this value normalized to 9,540 μL and 7,570 μL by the first and second follow-up tests, respectively. The diastolic blood pressure of the assistant was 90 mmHg at the initial checkup, but normalized by the follow-up tests. At the first follow-up, urine lead levels in the primary operator and assistant were 27.57 μg/g and 125.6 μg/g of creatinine, respectively; both values fall below the threshold of the recommended biological exposure index of 150 μg/g of creatinine.

**Table 3:** **Clinical laboratory results**

	**Primary operator**	**Assistant operator**
Blood lead level (μg/dL)		
Initial sampling (Dec. 24, 2013)	61.1	51.7
1st follow-up sampling (Jan. 16, 2014)	63.1	68.8
2nd follow-up sampling (Feb. 21, 2014)*	40.8	45.2
3rd follow-up sampling (Mar. 24, 2014)*	39.0	44.7
4th follow-up sampling (Apr. 24, 2014)*	42.0	-†
Blood analysis (at initial sampling)		
Hemoglobin (g/dL)	15.8	16.3
Hematocrit (%)	46.4	47.6
RBC (×10^3^/μL)	5.39	5.56
WBC (×10^3^/μL)	5.68	10.09
Platelet (×10^3^/μL)	227	245
Creatinine (mg/dL)	1	0.89
Urinary analysis (at 1st follow-up sampling)		
Urine lead level (μg/g creatinine)	27.57	125.6
β2-microglobulin (mg/day)	-	0.04
Blood pressure (at initial sampling)		
Systolic blood pressure (mmHg)	130	120
Diastolic blood pressure (mmHg)	70	90
Blood cell morphology		
1st follow-up sampling (Jan. 16, 2014)	Slight platelet aggregation	-
2nd follow-up sampling (Feb. 21, 2014)*	Slight platelet aggregation	Normal
3rd follow-up sampling (Mar. 24, 2014)*	Normal	Normal
4th follow-up sampling (Apr. 24, 2014)*	Normal	-†

*After job reallocation.

†The assistant operator left the company before the fourth follow-up.

The white blood cell differential counts for both workers were within the normal range. The results of a blood cell morphology test in the primary operator showed slight platelet aggregation at the first and second follow-up tests, but this value normalized by the third and fourth follow-up tests. However, the blood cell morphology test results for the assistant remained normal. The primary operator had an elevated gamma-glutamyl transpeptidase level (57 U/L) at the initial health checkup, but this value normalized in the follow-up tests reaching 42 U/L by the third follow-up.

After the first follow-up, the BLL of three workers in the metalworking process (two workers in charge of the machining center tooling process and one worker in charge of the CNC process) and the on-site supervisor were also measured. Their BLLs were 22.66 μg/dL, 29.28 μg/dL, 20.98 μg/dL, and 26.70 μg/dL, respectively, with an average BLL of 24.91 ± 3.78 μg/dL.

### Psychological tests

Elevated BLL is associated with psychological symptoms such as depression, anxiety, cognitive deficit, and memory loss [4,15]. As such, psychological tests were performed by trained psychologists at the first follow-up. These tests included a digit span to evaluate attention, the Seoul Verbal Learning Test to evaluate verbal memory, a verbal fluency test, the Stroop test, a psychomotor speed measurement, a working memory test using the Korean-Wechsler Adult Intelligence Scale to evaluate frontal lobe function, the Korean version of the Boston Naming Test to evaluate language skills, the Rey-Osterrieth complex figure test to evaluate visuospatial skills, and the Hamilton Depression Scale and Beck Depression Scale to evaluate mood status. Slightly reduced psychomotor speed on the Korean-Wechsler Adult Intelligence Scale (raw score 41/135, percentile score 5) and a reduced interference score in the Stroop test (interference score 11, percentile score 10) were noted in the primary operator; however, all other results were normal.

### Nerve conduction test

The primary operator complained of weakness while using chopsticks at the fourth follow-up; therefore, a nerve conduction velocity test was performed by a trained neurologist at the fourth follow-up. Results were consistent with carpal tunnel syndrome and included decreased sensory nerve action potential, slow sensory nerve conduction velocity in the forearm (wrist and palm) wrist segments of the right median nerve, prolonged terminal latency, decreased compound muscle action potential in the wrist (elbow and elbow) axilla segments of the right median nerve, and a prolonged F-wave in the right median nerve. Nerve conduction tests were not performed in the assistant because he left the company before the fourth follow-up.

## Conclusions

This report documents two cases of high lead exposure in workers from a leaded bronze ingot foundry. In 1978, lead poisoning was reported in a major smelting factory located in Janghang, South Korea. Severely lead poisoned workers from this incident were admitted to the hospital to chelate the lead and lower their high BLLs [7]. Soon after, in October 1983, lead poisoning was reported in the Banwol Industrial Complex in South Korea. One hundred and one lead workers in seven factories that handled lead daily (six litharge-making factories and one storage-battery factory) were investigated. Among these, 33 cases of lead poisoning (BLL >80 μg/dL) and 36 cases of high lead absorption (BLL >60 μg/dL) were diagnosed [8]. In September 1986, 66 out of 258 lead workers in a storage-battery factory in South Korea were screened for lead poisoning and referred for laboratory tests; 29 of these workers were diagnosed with lead poisoning and 12 had severe lead poisoning that required hospital admission to chelate the lead and lower their BLLs [9].

The most important aspect in treating lead poisoning is eliminating the source of lead exposure. However, eliminating the source of lead can be very difficult depending on the socioeconomic challenges involved. The Occupational Safety and Health Administration and Association of Occupational and Environmental Clinics have published guidelines of the clinical symptoms of lead poisoning to facilitate diagnosis and set standards for its management in suspected cases of lead exposure [5,16]. In general, BLL is used as an important biological indicator in clinical evaluations, worksite monitoring, and public health.

Shortly after lead is inhaled or ingested, it can enter the bloodstream and travel to soft tissues. After several weeks, the absorbed lead can be deposited and stored in mineralizing tissues such as bones. In the current study, we were unable used X-ray fluorescence to measure the workers’ long-term lead exposure. With X-ray fluorescence, we hypothesize that the bone lead level of the primary operator was higher than that of the assistant, considering his long exposure. Lead can also interfere with heme biosynthesis by inhibiting *d*-aminolevulinic acid dehydratase and ferrochelatase activity, thus increasing blood and plasma d-aminolevulinic acid and free erythrocyte protoporphyrins [17], which leads to anemia. In the primary operator and assistant, hemoglobin levels were 15.8 g/dL and 16.3 g/dL, respectively, which are both within the normal range; thus, it was assumed that lead exposure did affect the enzymatic heme pathway of these two workers. Lead poisoning may also result in abdominal pain, loss of appetite, vomiting, nausea, constipation, and/or diarrhea. No specific symptoms or signs were found in the review the patients’ history or during the physical examination. Lead poisoning can also cause peripheral nerve damage that causes muscle weakness. In addition, behavioral changes vision and hearing impairment are possible. At very high BLLs, lead can affect the brain causing convulsions, a coma, and even death [18]. Moreover, lead can inhibit the N-methyl-D-aspartate receptor that is related with hippocampus-mediated learning and memory resulting in defects in the memory processes of the brain. Lead can also cause deficits in neurotransmission, disrupt neurotransmission and intracellular Ca2+ dynamics, and ultimately result in neurotoxicity [19]. Among the current cases, the primary operator showed a slightly reduced psychomotor speed and interference control during psychological assessment and had a right median nerve abnormality that is consistent with carpal tunnel syndrome.

Clinical symptoms of lead poisoning are typically associated with chronic lead poisoning; thus, cases of acute lead poisoning are difficult to diagnose. In certain cases, lead poisoning can manifest asymptomatically [20]. In the present cases, aside from subtle neurologic abnormalities in the primary operator, no specific clinical findings of lead poisoning were evident for either of the workers. Although we cannot eliminate the possibility that the aforementioned abnormal findings in the primary operator were the result of chronic lead exposure, the symptoms were not severe. Moreover, the associations between reduced motor conduction, short-term memory loss, and impaired visual-motor coordination with lead poisoning have not been evaluated [21]. Although our subjects had no clinical symptoms, subclinical changes such as behavioral changes, subclinical hypertension, or a subclinical tumor might have existed.

The assistant presented with a higher BLL than that of the primary operator at all follow-up evaluations, but not at the initial assessment. In the workplace, the assistant continuously stood in front of the molding frame during the molding frame preparation with his head bent towards the frame; therefore, his mask was in close contact with the molten metal and it was assumed that he inhaled a greater proportion of the lead fumes. Furthermore, the level of work experience might have influenced the differences in the BLLs between the primary and assistant operators. In addition, although an exhaust system was installed above the furnace, no exhaust system was present above the molding frame; thus, these workers were continuously exposed to lead fumes while transferring the molten metal during the casting process.

After the first follow-up, the primary and assistant operators had BLLs of approximately 40 μg/dL and were relocated the CNC department. Nevertheless, the BLL of the other CNC workers were found to be >20 μg/dL, which is much higher than the average in South Korea of 1.77 μg/dL that was reported in 2011 [21]. These findings suggest the possibility of background lead exposure at the work site. The smelting and casting processes and CNC/machining center tooling processes were separated by a wall; therefore, the BLLs of employees working in processes other than smelting likely did not become exposed to lead from the smelting process.

Metal cutting occurs at temperatures between 214°C–594°C [22]. As such, lead fumes caused by sublimation can also cause health damage in high-temperature cutting operations [16]. It is possible that the BLL of workers in other processes increased because of these lead fumes. Another possibility is the absorption of lead through the digestive tract and skin. One expended furnace had been left in the factory, and sand was strewn on the floor of the work site. We measured a lead content of >10% in the expended furnace and sand. Lead in these items may have contaminated the workers’ hands, clothes, and any drinks consumed in the workplace. Relevant studies have reported that 5%–10% of the lead absorbed through the digestive tract is ultimately absorbed by the human body [23]. Therefore, workers at the present site may have absorbed lead not only through the respiratory tract but also through the skin or mouth.

The atmospheric lead concentration in this work environment was less than the Ministry of Labour standard (0.05 mg/m^3^), but more than the Korean environmental atmospheric standard (0.0005 mg/m^3^) and EPA standard (0.00015 mg/m^3^) [24,25]. Since the atmospheric lead concentration does not reflect skin absorption, bodily accumulation, or personal hygiene maintenance, the correlation between the atmospheric lead concentration and BLL is considered weak [26]. In the present cases, the atmospheric lead concentration does not reflect the skin and oral exposure to lead dust at the work site. In addition, exposure to high concentrations of lead fumes during smelting work is assumed to occur while pouring molten metal into molding frames. Such tasks occur over a relatively short time (5–10 min per session, 5–6 sessions per day). Work environment measurements are collected as the mean lead exposure over 8 h; thus, this measurement may not have accurately reflected their instantaneous exposure to high lead concentrations. In addition, during the 8-h measurement period, the number of actual work sessions may have affected the measured results.

In the present work site, the lack of the MSDS for the primary material was substituted with an inspection certificate. Nevertheless, an MSDS of the reducing agents were not provided. Moreover, despite the fact that the furnace process requires a level 1 dust mask, workers at this company were only provided with level 2 dust masks. In addition, protective gear was worn inconsistently [27,28]. The work site near the furnace was also strewn with waste, such as that from the expended furnace, and high concentrations of lead were detected in this waste. Furthermore, exhaust systems were not properly installed at the work site.

Taken together, to help prevent lead exposure, all companies must be equipped with an MSDS for all materials used in their manufacturing process, and the owners and workers of these companies must all be aware of the contents of the MSDS. Furthermore, to minimize lead exposure, appropriate protective gear, including level 1 dust masks and protective goggles, should always be provided and worn, and an appropriate exhaust system must be installed. In addition, to eliminate contamination by lead dust, the work site should be cleaned daily with a vacuum cleaner or water. Smoking and eating/drinking should be prohibited at the work site to protect the workers, and facilities that promote personal hygiene by including sinks, baths, and washing machines should be made available. In addition, face washing and bathing should be mandatory after daily work. Employees should also be educated on the above materials, and any notifications regarding handling precautions and personal protection should be displayed in an easily visible location. Moreover, work environment measurements and specialist checkups should be conducted regularly to monitor the degree of lead exposure among workers. In cases of high lead exposure, appropriate intervention methods should be immediately performed to protect the health of workers [28].

Because our report only conducted follow-ups and observed two workers with high lead exposure after an occupational health checkup, we could not adequately investigate the source of lead exposure with respect to the entire work site or compare the lead exposure throughout the facility. Thus, future epidemiological studies on the workers at the present work site are needed.

To our knowledge, this is the first report of a high lead exposure in a leaded bronze ingot foundry in South Korea. Although the two workers studied here did not demonstrate clinical symptoms of lead poisoning, the BLLs of these workers did reach a maximum of 69 μg/dL, and even the other workers at the metalworking process, which were isolated from the foundry, exhibited a relatively high mean BLL of 24.91 ± 3.78 μg/dL. To prevent lead exposure among employees in smaller factories, appropriate periodic occupational health checkups and functional environment protection measurements to protect the workers’ health are needed.

## Consent

We performed a retrospective chart review and collected no personal identification data. The Institutional Review Board of Inje University Busan Paik Hospital approved our waiver of written informed consent and review exemption (IRB No. 14–0115).
